# The Week's Work

**Published:** 1910-03-26

**Authors:** 


### The Week's Work.

### WOOD FLOORING AND OILING.

The question of floor material, as was evidenced by the recent discussion in the "Question Box," is one on which a legitimate difference of opinion may be held by hospital workers. Those who are interested in it will, however, find some useful information on the subject in a recent series of official memoranda emanating from the German Education Department. Some years ago, we believe, the Prussian Government instituted an inquiry into the hygienic conditions under which scholars in the elementary schools were being educated, and this inquiry was followed by an interesting report, in which the question of flooring was touched upon. It was noticed, for instance, that in schools where the flooring was of uncovered wood, the percentage of dust in the air was far larger than in rooms where linoleum was being used, and as a result of this observation experiments were made with the object of finding out in what manner it was possible to "lay" this excess of dust by employing dustless oils. These experiments were followed with much interest by the designers of new German hospitals, who were by no means convinced that the tiling methods employed in the more modern institutions were unqualified successes, but who have hitherto objected to wood flooring on the ground that it cannot be kept clean without a great deal of trouble, and that the usual method of oiling causes a certain musty smell which is highly obnoxious in a hospital ward. The experiments proved that if oiling is properly carried out—as, for instance, it is in most English hospitals—the results are thoroughly satisfactory. The Education Department has recently issued a memorandum which contains the latest conclusions of the expert committee appointed to investigate the matter.

### DUSTLESS OILS.

The memorandum recommends that the dustless oils be used upon either hard-wood or soft-wood flooring. It is imperative that the flooring must be well laid, free from cracks, and of properly-seasoned wood. The floors must be thoroughly scrubbed with soap or soda and water and then carefully and methodically dried before the oils are applied, and the oiling must be repeated once or twice yearly, according to the amount of wear. The oil is spread evenly and in a thin coating by means of a felt rubber, and usually dries very quickly, leaves no smell, and is non-slipping. The memorandum does not advise that such floors should be washed once they have been oiled; dry mopping is quite sufficient to clean them. This may be true in the case of schools, but is certainly not to be advised in the case of wards in hospitals or other institutions. Such oiled floors easily bear scrubbing with soft soap and water, and the possible objection to this method of cleaning—that it renders the oiled floor more slippery—is not well founded, and is probably only true in the case of floors that have been badly or imperfectly oiled. Slipperiness is not uncommon when hard-wood flooring, such as oak or teak, is oiled, and is, of course, found in every case where the oiling is quite fresh; but a well-oiled soft-wood floor is certainly not so slippery as the tiled, or, worse still, the glass floor which is found in some theatres.

### THE REGISTRATION OF NURSING HOMES.

At the annual meeting of the Home Hospitals Association last week, Sir Henry Burdett referred to the insanitation, overcrowding, and high charges of private nursing homes, and stated that there is no possibility of protecting the public against these abuses until and unless a system of registration is instituted. While Fitzroy House is a splendid example of what a home hospital should be, it is unfortunately true that a large percentage of private nursing homes in this country are by no means models of comfort, and present many features which are amazingly reminiscent of the days of Howard. This is a fact which is perfectly well known to most members of the profession who patronise these homes: it is, unluckily, not one that the public has grasped, else State interference and ultimate registration would no longer be merely pious hopes but actualities. In Australia the Government has already taken action in the matter; in Germany, where there are more private hospitals and nursing homes than in any other country perhaps, there is a tendency towards similar legislation, and at all events there already exist certain safeguarding rules which make it impossible for a German private clinic to be a death-trap to a certain class of patient. In this country, however, there are no safeguards. Everyone and anyone can establish a private nursing home, and so long as it is not so execrably insanitary as to necessitate a visit from the local health authorities, it is allowed to remain and to entice patients from the public.

### THE PROFESSION AND NURSING HOMES.

The practitioner who patronises these homes is forced to do so in most cases owing to the fact that they appeal to him for the moment, when he thinks that his patient must have the benefit of the skilled nursing that is provided, even at the risk that the surroundings and environment are not all that they should be. When he sends his second patient he hopes that the faults which he pointed out at his last visit have been rectified. Probably they have, but in the meantime other patients have suffered, and there are many cases in which no attention whatever has been paid to the remonstrances of surgeons and medical practitioners with regard to the sanitation and internal arrangements of these homes. At the present moment the profession is powerless to cope with the abuses which exist in such homes. There is always the fear that any organised opposition may be looked upon as a flagrant attempt at advertising, while it is impossible for the average surgeon, in present circumstances, to make a separate but effectual protest. What is wanted is that the public as a whole should be roused to a sense of the gravity of these abuses. Once that has been done it will be comparatively easy to get the profession, with the public at its back, to deal with them, and to enable a scheme for the registration and inspection of these institutions to be carried through Parliament.

### THE INFANTS' HOSPITAL.

The annual report of this institution is an interesting and well got up pamphlet, well printed, and exceedingly well illustrated with reproductions of photographs showing various phases of the life in the wards. These are points which those who are concerned in drawing up the annual report of a hospital should always bear in mind. We have on more than one occasion pointed out that institutions do damage to their reputation, and, if they do not go so far as to alienate the sympathies of readers of these reports, at least do not make any great effort to stimulate such interest, by issuing bald, uninviting, and uninteresting annual reports. The Infants' Hospital is comparatively young yet, and its energy is praiseworthy. We are glad to see that its activities are increasing. Owing to the inability of the Committee to arrange a special appeal last year, the necessary funds to meet expenses of maintenance were not obtained, and there is, consequently, a deficit of £450 at the end of the year. This, we have no doubt, will be extinguished very soon, for the friends of this charity have shown themselves active and energetic, and we are glad to note that the Committee has every reason to anticipate the establishment of an adequate endowment fund. The King's Fund made a first grant of £25 to the hospital last year, and the other funds have generously contributed towards the finances. We think the Committee should take steps to make it more generally known that this institution is open to post-graduate students, and that special classes, both clinical and pathological, for qualified medical men—not for students—are held from time to time. If this were more generally known we feel sure that the hospital would receive increased support from the profession and the public alike.

### THE DRUG MARKET.

As is usual on the eve of a public holiday, business in drugs and chemicals is extremely dull and few changes in price have taken place during the past week. Although there has been a tendency to rather lower prices in the market for crude glycerine, the price of medicinal quality still remains at the high figure which has been quoted for the last few months, and no reduction in this price is to be made, at any rate at present; this was decided upon at the annual meeting of the principal international glycerine refiners which was recently held in London. Cream of tartar is a shade dearer. Ipecacuanha tends to advance in price rather than to decline. In a recent issue of The Hospital, under the above heading, readers were reminded of the uncertainty in the immediate future of the price of spirit owing to the unsettled political situation and the delay in finance legislation, and it was pointed out that no advantage would be gained by making purchases of spirit to cover more than present needs. It is now believed in many quarters that, even in the event of the present Government remaining in office, the extra duty of 3s. 9d. per gallon on proof spirit proposed in the Budget for the year ending March 31, 1910, would be remitted in the Budget for the year ending March 31, 1911. Should this prove to be correct, there would, of course, be a corresponding reduction in the price of spirituous preparations, and it would be wise to refrain from purchasing such preparations in greater quantity than is necessary for, say, three or four weeks' requirements.

